# Primary and Secondary Symptoms and the Dose

**Published:** 1891-02

**Authors:** Charles B. Gilbert

**Affiliations:** Washington, D. C.


### PRIMARY AND SECONDARY SYMPTOMS AND THE DOSE.

CHAS. B. GILBERT, M. D., WASHINGTON, D. C.*

[The following paper seems to bear on the question asked by our venerated "S. L." in THE HOMŒOPATHIC PHYSICIAN for December, 1890, and is offered for what help the facts stated may be in solving that difficult question.]

In the *Medical Advance* for January, 1886, after reporting a case which brought up the question of primary and secondary symptoms, I wrote as follows:

"The bane of our materia medica is the incorporation of secondary symptoms; these are not directly due to the action of the drug, but are the result of the reaction of the system against the poison. A homœopathic prescription cannot be made on the secondary symptoms of a proving, because they are not the symptoms of the drug disease, but evidence of the reactive power of the organism in the direction of health, and hence have no simile in diseased states of the body." This I will take as my text.

By primary symptoms are meant those first appearing as the result of the action of a medicinal substance upon any tissue. In fatal cases of poisoning there are *only* primary symptoms, and death follows because the secondary symptoms are lacking; the secondary symptoms then must be the result of reaction which must and can come from the system only; that these secondary symptoms are not due to an opposite action of the chemical substances is evident; for no substance in its action contradicts itself; chemical laws are God's laws: does Corrosive Sublimate ever cease to be a corrosive irritant? do acids ever tire of acid action and become alkaline or even neutral? Does the chemistry of the body go on under any different laws or its laws turn upon themselves?

It may be urged that remedies in large doses act in one direc-

---

*Condensed from a paper read before the Washington Homœopathic Medical Society of District of Columbia, in November, 1890.

tion, while in small doses they act in another, as claimed by the old school; no symptoms can be exhibited as the result of drug action until the nervous system has been irritated, the first action of which must be a giving way; but if the onset is not too fierce, the system rallies to repel the invader, the reaction being governed in time and degree by the strength of the dose: if a small, or so-called tonic-dose be given, it must be so small that the system can rally with little delay to repel it, while if a larger, or so-called depressant dose be given, the vital force is overcome by it for a longer or shorter period according to the strength of the dose. In diseased conditions this is worse, for inasmuch as any dose, given homœopathically, will act in a direction similar to the disease, the system must overcome both drug *and* disease. Just as the plumb-line swings back beyond the centre in such proportion as it has been pushed away from it, minus the effect of gravity and friction, so the vital force will carry the reaction of the system beyond the healthful equilibrium to the same extent that it has been forced away from it, minus our inherent power to preserve health; all have seen many examples of this.

Are we then to take the evidences of a systemic struggle against its foe as indications for a new and stronger dose of the remedy which was as a whole or a part of the prime cause of the excessive reaction?

Prof. E. M. Hale, several years ago, propounded a law of dose which the writer never admitted, but denied, in the *Advance* in 1886: "Primary symptoms call for high attenuations, while secondary symptoms call for low attenuations." The first part, the writer's experience has proven to be true, the latter, false.

Let us suppose a patient to have had arsenical diarrhœa, and to have been cured with Arsenic$^{30}$, the attack being followed by a severe reaction of the system for which he goes to Dr. No. 2, who says, "This patient has the secondary symptoms of Arsenic." If he shall give him Ars.$^{30}$, will he not be adding force to the medication already received and so continue the violent reaction still longer until the system again swings the other way through exhaustion of its irritability? but if the doctor shall give a dose strong enough to neutralize the over-reaction of the system against the disease, it would be substituting a drug action for a healthful action; this would be followed by new reaction of the system against the drug, and so on, the patient losing strength all the while; but if the new prescription shall be of a remedy whose primary action is similar to the over-action of the system and opposite to the primary action of the Ars., and if only such a dose be given as will arouse just sufficiently new reaction of the system in an opposite direction to the reaction against the Ars., then will health be restored quickly, pleasantly, and permanently, because the reaction of the system will not be toward the drug, but toward health, while, in response to a strong dose of Ars., it would go away from healthful action and toward that of the drug.

As an example, let us take the case of a man who, after an Ars. poisoning, had obstinate constipation for two years. Would any one think of trying to cure him with tangible doses of Ars.?

Or if a patient should present himself with a primary constipation, resembling the reactive effect of Ars., is it not evident that a strong dose of Ars. would be required? And this would be an action toward the drug, and hence but palliative.

Suppose that instead of giving the thirtieth of Arsenic, in the first place a dose be given sufficiently large to effect a hearty person, say the 3x. If the 3x will make a well person sick, how much more easily will it affect a sick person? It is evident, therefore, that the dose for primary symptoms must be reduced below the sick-making power in the healthy, just in proportion as the resistance of the vital force against disease action has been reduced below the normal. Let us suppose the dose to have been reduced below the sick-making power far enough to allow reaction expressed by 1. If the dose shall be reduced farther the reaction of the vital force may be expressed by 2; if still farther by 5, 10, 50, 100, 1,000, and soon up to a point where the vehicle fails to contain any Arsenic power.

Let us look at another familiar remedy, which probably has been more abused in its symptoms of the bowels than any other, viz.: Sulphur. The provings and the poison records as well as the *United States Dispensatory* show that its primary effect is as a laxation from the watery stool, that drives one out of bed early in the morning to a pasty stool, according to the size of the dose. We find also, "stool hard as if burnt," as given by Hahnemann, but, owing to a fatal defect in his *Materia Medica* we have no means of knowing whether it was primary or secondary. We find, in the other provers, however, that those who suffered from constipation did so either after having diarrhœa, or else as a result of high attenuations used in proving.

On the contrary, as to Nux-vomica, Hahnemann says, in a foot-note, what can be corroborated by any physician: "Persistent, profuse diarrhœa-like stool, which constitutes true diarrhœa, never, so far as I have observed, occurs in the primary action of Nux-vom., and the diarrhœa expressed in this symptom consists of very small stools, mostly of mucus, accompanied by straining, or, when the evacuation is copious, and then, it is the secondary action. The curative effect in a patient who has previously suffered from constipation, *ineffectual desire for stool.*"

This constipation resembles the constipation after the diarrhœa of Sulphur. As was found under Sulphur, so we find under Nux-v. that the high attenuations produce upon the system symptoms like those produced by the vital force, in reacting against the crude doses, for instance, the following: "Exceedingly sudden attack of diarrhœa at night, when least expected. He had to get out of bed and run for his life; no premonitary symptoms whatever" (1,000 attenuation), under Sulph., from the same attenuation. "Feeling of great constipation and hardness in the bowels." In another prover, extreme constipation. From this it would seem that of the secondary symptoms of Nux and Sulph. each resembles the primary symptoms of the other, and that we may be justified in saying that those remedies that in their primary effects resemble the reaction of the symptoms against the previous remedies will follow them homœopathically; we may also say this: If a high attenuation will produce symptoms in a well person that are *contrary* to the recognized effects of the drug and similar to the reaction by the system against the drug itself, does not our duty to our art require, that in selecting the dose we shall select such a one as will not aggravate the symptoms of the disease, in order to lead to reaction by the system (the so-called secondary symptoms), but rather one just sufficient to enhance directly, and as far as possible, reaction in the system without such aggravation? That Hahnemann had some such idea in regard to the size of doses as compared with the strength of the vital force, is shown by his prescribing for the washerwoman. He says, "As the woman was very robust, and as the forces of disease had affected her organism so painfully that she was not able to continue her work, and as, moreover, her vital powers were unimpaired, I gave her a full drop of the tincture of Bryonia with directions to see me again in forty-eight hours. I told my friend E., who was present, that the woman's health ought to be restored after this period, which he doubted, not being yet fully converted to the new doctrines" (*Mat. Med. Pura*).

As to another case—a man to whom he gave Puls.—he says: "Being weak and worn out, he only took half a drop of the sixteenth potency of Puls. toward evening."

In the first case he shocked a strong vital force with a single dose, which, not being repeated, left the system free to react as sharply as possible against this artificial irritant; the result was that not only the strong woman in the first case was well the next day, but the weaker man also.

May we then venture to define a "high attenuation"? A "high attenuation" of a remedy is one that will produce upon the system symptoms that are contrary to the action of the crude drug, or attenuations which, in their action, are similar to the crude drug. Of course in the sick, who are thus made more sensitive to the action of medicine, homœopathically, a smaller dose must be given than would be required to affect the well in a similar manner, and must be left to the judgment and experience of the physician.

Accepting the foregoing views as true, it is evident that to prescribe for constipation, Agar., Cocc., Gratiola, Laur., Petrol., Ratanhia, Senega, Sulphur, and above all Verat-alb., or to prescribe Bry., Nux-v., Kali-c., Lyc., Nat-m., Nit-acid, Sepia, Silic., as given by Bell, for diarrhœa can but be palliative, and allows the system to recover by action of the vital force.

There is another point in connection with this matter. Dr. C. Hering says that in a proving the last symptoms to appear are the most characteristic; not the so-called secondary symptoms, but those symptoms which while they come late are the first evidence of an attack upon that particular part of the system: the explanation of this would seem to be that inasmuch as nature's strongest parts and the last to yield are the vital centres and as those most easily affected are the more external functions, that therefore those are first affected which are most easily disturbed, and being more easily disturbed are more apt to be affected by various but similar remedies in apparently much the same way in consequence; but the more interiorly the action goes, the nearer it approaches to the vital force, the nearer it approaches that which individualizes the patient; those symptoms, therefore, which are individualized by the patient are the most characteristic; hence, as Hahnemann taught, the *mental* symptoms are the most important of *all*, and must be covered by the curative remedy, for the mind lost, *all* is lost save mere animal life, which is not much more than vegetable life.

It may be said that the provers are not unanimous always in their reports of the effects of a remedy upon them; this is quite true; suppose two persons one of whom has a tendency to constipation, the other to diarrhœa, whose health is disturbed; give both Sulphur in a mild dose, can it be supposed that both would be affected alike? Assuredly not. Often it is stated that such and such a condition to which the prover had been subject disappeared during the proving, and has not returned. That prover would of course have been affected by the remedy in a different direction from a prover whose tendency was the other way or who even was well as to that function. The combined testimony of provers is necessary to make the picture perfect.

The points I wish to emphasize are these:

1. "Primary symptoms" only are indications for the selection of a remedy, as taught by Hahnemann.

2. There are no "secondary symptoms" of a remedy, but such so-called symptoms are evidences of the reaction of the system.

3. Remedies follow each other, homœopathically, in which the action of the second is similar to the reaction against the first.

4. The dose must be reduced below the sick-making power until it is capable of inducing action in an opposite direction to the effect of crude drug upon the well without any appreciable aggravation of the symptoms, and this constitutes a "high attenuation."

5. The dose for the sick must be smaller (higher) than that required to produce the required re-action in the well.

