# Corrections

**Published:** 1891-03

**Authors:** 


CORRECTIONS.—February No., page 63, fourth line from top, for laxation read *laxative;* page 63, nineteenth line from top, for then read *thin;* twentieth line from top, after "constipation" insert *with.*

